# Retinal Microvascular and Peripapillary Profiles in Mild Autonomous Cortisol Secretion Versus Nonfunctioning Adrenal Adenomas

**DOI:** 10.3390/jcm15207850

**Published:** 2026-10-12

**Authors:** Selin Şimşek Alkan, Aslı Vural, Vildan Gürses Beşli, Sibel Zırtıloğlu, Seda Karslı, Seda Turgut, Didem Acarer Bugün, Naim Pamuk, Hamide Pişkinpaşa, Sema Çiftçi

**Affiliations:** 1 Department of Ophthalmology, Faculty of Medicine, Biruni University, 34010 Istanbul, Türkiye; 2 Department of Ophthalmology, Bakırköy Dr. Sadi Konuk Training and Research Hospital, University of Health Sciences Türkiye, 34147 Istanbul, Türkiye; asli.deger@hotmail.com (A.V.); vildangrss@gmail.com (V.G.B.); 3 Department of Ophthalmology, Kanuni Sultan Süleyman Training and Research Hospital, University of Health Sciences Türkiye, 34303 Istanbul, Türkiye; sibel1077@hotmail.com; 4 Department of Endocrinology and Metabolism, Bakırköy Dr. Sadi Konuk Training and Research Hospital, University of Health Sciences Türkiye, 34147 Istanbul, Türkiye; sedakarsli36@gmail.com (S.K.); seda.dr@gmail.com (S.T.); didemacarer@hotmail.com (D.A.B.); naim814@hotmail.com (N.P.); h_karagoz@hotmail.com (H.P.); sdogansen@gmail.com (S.Ç.)

**Keywords:** mild autonomous cortisol secretion, adrenal incidentaloma, subclinical hypercortisolism, optical coherence tomography angiography, deep capillary plexus, retinal nerve fiber layer, vessel density

## Abstract

**Background/Objectives**: Mild autonomous cortisol secretion (MACS) is associated with increased cardiometabolic morbidity, but its relationship with the retinal microcirculation is unknown. We compared retinal and peripapillary microvascular parameters between MACS and nonfunctioning adrenal adenoma (NFA) and, secondarily, examined their associations with continuously modeled post-dexamethasone cortisol. **Methods**: In this single-center, retrospective, cross-sectional study, 98 patients (45 NFA, 53 MACS) were included, and optical coherence tomography angiography (OCTA) and structural OCT data from one eye per patient were analyzed. The primary outcome was whole-image deep capillary plexus (DCP) vessel density. Multivariable models were adjusted for age, sex, body mass index, and cardiometabolic comorbidities; secondary analyses were corrected using the Benjamini–Hochberg false discovery rate (q values). **Results**: OCTA was evaluable in 89 patients. The primary outcome did not differ significantly between groups in the main analysis (NFA vs. MACS, 48.64 ± 5.71% vs. 47.31 ± 5.77%; adjusted difference −1.29 percentage points, 95% CI −3.97 to 1.38; *p* = 0.339) or in any sensitivity analysis. In secondary analyses, superior retinal nerve fiber layer (RNFL) thickness was greater in MACS (β = 17.38 µm; q < 0.001) and increased with ln(post-dexamethasone cortisol) (β = 13.74 µm; q = 0.010). Higher ln(post-dexamethasone cortisol) was also associated with lower parafoveal DCP (β = −2.72 percentage points; *p* = 0.013) and inside-disc radial peripapillary capillary vessel density (β = −3.13 percentage points; *p* = 0.026), which did not survive multiplicity correction; no superficial capillary plexus sector was associated with either exposure (all *p* ≥ 0.37). **Conclusions**: MACS was not significantly associated with whole-image DCP vessel density. The secondary associations with continuous cortisol are cross-sectional and hypothesis-generating and require prospective confirmation.

## 1. Introduction

Adrenal incidentalomas are adrenal masses detected unexpectedly during imaging performed for reasons unrelated to suspected adrenal disease. Although most of these lesions are benign and hormonally inactive, a subset of patients exhibits autonomous cortisol secretion without the typical clinical features of overt Cushing syndrome. According to the current European Society of Endocrinology guideline, mild autonomous cortisol secretion (MACS) is defined by a post–1 mg overnight dexamethasone suppression test (DST) serum cortisol concentration greater than 1.8 µg/dL (50 nmol/L) in patients without clinical features of overt Cushing syndrome [1]. Compared with nonfunctioning adrenal adenomas (NFAs), MACS has been associated with a higher prevalence of cortisol-related comorbidities, including hypertension, type 2 diabetes mellitus, dyslipidemia, cardiovascular disease, and fragility fractures [1,2]. Cohort data also suggest that mortality risk may increase with higher post-dexamethasone cortisol concentrations [3]. These observations indicate that low-grade but chronic cortisol excess may exert target-organ effects even in the absence of an overt Cushing phenotype.

Vascular and microvascular dysfunction may represent one pathway linking MACS to its cardiometabolic burden. Glucocorticoids can influence vascular tone, nitric oxide bioavailability, oxidative stress, and endothelial cell survival, and experimental models of glucocorticoid-induced hypertension have demonstrated endothelial cell loss and microvascular rarefaction [4,5]. However, hypertension, diabetes mellitus, obesity, and dyslipidemia, which frequently coexist with MACS, may independently affect the microcirculation. Consequently, the magnitude and direction of the independent association between mild cortisol autonomy and human microvascular alterations remain uncertain.

The retinal and optic nerve head circulations provide a unique opportunity for non-invasive assessment of the microvasculature. Optical coherence tomography angiography (OCTA) enables depth-resolved, quantitative visualization of retinal and peripapillary microvascular networks without intravenous dye administration [6]. It permits evaluation of the superficial capillary plexus (SCP), deep capillary plexus (DCP), foveal avascular zone (FAZ), and radial peripapillary capillary (RPC) network, while structural optical coherence tomography provides complementary measurements of retinal nerve fiber layer (RNFL) thickness. OCT and OCTA may therefore be suitable for investigating potential subclinical ocular target-organ involvement associated with MACS.

Existing ocular imaging evidence in hypercortisolism has predominantly focused on overt Cushing syndrome and the choroid. Increased choroidal thickness and a higher prevalence of pachychoroid spectrum disease have been reported in patients with active Cushing syndrome, and choroidal thickening correlates with the degree of cortisol excess [7,8]. Two studies have examined the eye in mild cortisol excess, both confined to the choroid: one reported greater subfoveal choroidal thickness and a higher prevalence of pachychoroid features in MACS than in nonfunctioning adenoma [9], whereas a pilot study using enhanced-depth-imaging OCT and choriocapillaris flow analysis found no difference from healthy controls in choroidal thickness, choroidal vascularity index or choriocapillaris flow and no correlation with any hormonal parameter [10]. Retinal capillary plexus and peripapillary measurements in this population have so far been reported only in abstract form [11]. To our knowledge, the present study is the first full-length report of retinal and peripapillary microvascular parameters in mild autonomous cortisol secretion and the first to model post-dexamethasone cortisol as a continuous exposure.

In this retrospective cross-sectional study, we aimed to compare retinal microvascular and peripapillary structural characteristics between patients with MACS and those with NFA using OCTA and structural OCT. The primary outcome was whole-image DCP vessel density. Regional DCP and SCP vessel densities, FAZ metrics, RPC vessel density, and RNFL thickness were evaluated as secondary outcomes. As secondary, hypothesis-generating analyses, we additionally assessed associations between post-DST cortisol, modeled as a continuous exposure, and ocular parameters. Given the limited and directionally inconsistent evidence, a two-sided study hypothesis was adopted.

## 2. Materials and Methods

### 2.1. Study Design and Ethics

This single-center, retrospective cross-sectional comparative study was conducted in the Departments of Ophthalmology and Endocrinology and Metabolism at Bakırköy Dr. Sadi Konuk Training and Research Hospital, Istanbul, Türkiye. The medical records and ocular imaging results of adult patients evaluated for adrenal adenomas between 01/2020 and 06/2025 were retrospectively reviewed. The study was reported in accordance with the Strengthening the Reporting of Observational Studies in Epidemiology (STROBE) recommendations [12].

The study protocol was approved by the Clinical Research Ethics Committee of Bakırköy Dr. Sadi Konuk Training and Research Hospital (date: 19 June 2026; approval No.: 2026-14-19). The study was conducted in accordance with the principles of the Declaration of Helsinki. Written informed consent for the research use of previously collected clinical and ocular imaging data was obtained retrospectively from all included participants, and de-identified data were used in the analyses.

### 2.2. Participants and Endocrine Classification

Adults aged 18 years or older who underwent endocrine evaluation for an adrenal incidentaloma or adrenal adenoma were considered eligible. Final diagnoses of nonfunctioning adrenal adenoma (NFA) and mild autonomous cortisol secretion (MACS) were assigned by endocrinologists following evaluation of the clinical and biochemical findings.

In the absence of clinical features of overt Cushing syndrome, MACS was defined as a serum cortisol concentration >1.8 µg/dL (>50 nmol/L) following the 1 mg overnight dexamethasone suppression test (DST) [1]. Potential causes of false-positive DST results, including acute illness, physiological or psychological stress, interfering medications, and inadequate testing conditions, were assessed. When a potential confounding factor was identified, the 1 mg DST was repeated after correction. Plasma adrenocorticotropic hormone (ACTH) concentrations were evaluated to support ACTH-independent cortisol secretion and to exclude ACTH-dependent hypercortisolism. Patients with clinical features of overt Cushing syndrome or evidence of ACTH-dependent hypercortisolism were excluded. Other functional adrenal disorders, including pheochromocytoma and primary aldosteronism, were excluded by appropriate biochemical evaluation. Patients with a post-DST cortisol concentration ≤1.8 µg/dL and no evidence of hormonal hyperfunction were classified as having NFA [1].

Plasma ACTH concentrations were available for 52 of the 53 patients with MACS and 30 of the 45 patients with NFA, and the results of a 4 mg dexamethasone suppression test were available for 26 patients with MACS. All 52 patients with MACS who had a numerical post-DST cortisol result had a concentration above 1.8 µg/dL (range, 1.83–6.70 µg/dL), and all 44 patients with NFA who had a numerical result had values of 1.8 µg/dL or below (maximum, 1.73 µg/dL). Serum dexamethasone concentrations were not measured.

Participants whose numerical post-DST cortisol values were unavailable but whose final endocrinologist-assigned diagnoses were recorded were included in the categorical MACS–NFA comparisons and excluded from the analyses in which post-DST cortisol was evaluated as a continuous variable.

Patients with glaucoma, ocular hypertension, diabetic retinopathy, age-related macular degeneration, retinal vascular disease, or another ocular disorder that could affect retinal or optic nerve measurements were excluded. Additional exclusion criteria were systemic corticosteroid use, clinically significant media opacity compromising image quality, and a spherical equivalent refractive error outside the range of −3.00 to +3.00 diopters.

### 2.3. Ophthalmological Evaluation and Eye Selection

Best-corrected visual acuity, spherical equivalent, intraocular pressure, slit-lamp biomicroscopy findings, and dilated fundus examination findings were obtained from the ophthalmological records.

OCT/OCTA imaging was performed during the initial endocrine diagnostic work-up, on the day of referral from the endocrinology clinic or on the following day, before dexamethasone administration for the DST and before any adrenal-directed or cortisol-lowering treatment. Because imaging preceded dexamethasone administration, the DST-based classification was not available at the time of imaging, and image-quality screening and segmentation correction were also performed without knowledge of the endocrine diagnosis. For the present study, the previously acquired images and corresponding endocrine data of all patients referred during the study period were analyzed retrospectively.

Only one eye per participant was included to avoid statistical dependence arising from inter-eye correlation. The right eye was selected when both eyes were eligible; the left eye was included when the right eye was ineligible or had insufficient image quality. The participant was therefore the unit of analysis.

### 2.4. OCT and OCTA Imaging Protocol

Retinal and peripapillary imaging was performed using a spectral-domain optical coherence tomography angiography system (AngioVue; Optovue Inc., Fremont, CA, USA). The system operates at an approximate central wavelength of 840 nm and uses the split-spectrum amplitude-decorrelation angiography algorithm to detect variation between consecutive OCT signals caused by erythrocyte motion [13].

Fovea-centered 6 × 6 mm OCTA scans were used for macular microvascular assessment, whereas optic disc-centered 4.5 × 4.5 mm scans were used for optic disc and peripapillary assessment. En face images of the superficial capillary plexus (SCP), deep capillary plexus (DCP), and radial peripapillary capillary (RPC) network were generated using automated segmentation, which was checked and manually corrected when necessary.

Only images with a signal strength index of at least 7/10 and without clinically significant motion artifacts, decentration, or residual segmentation errors were included. Axial-length-based magnification correction was not performed because axial length measurements were unavailable.

### 2.5. Ocular Outcomes

The primary outcome was whole-image DCP vessel density. The primary analysis evaluated the difference in whole-image DCP vessel density between the MACS and NFA groups.

Secondary macular OCTA outcomes included foveal, parafoveal, and perifoveal DCP vessel densities and whole-image and regional SCP vessel densities. Foveal avascular zone (FAZ) area, perimeter, circularity and acircularity indices, vessel density in the 300 µm ring surrounding the FAZ (FD), and outer retinal flow area were evaluated as exploratory macular parameters.

Optic disc and peripapillary assessments included whole-image, inside-disc, peripapillary, and sectoral RPC vessel densities. Structural OCT parameters included average and sectoral retinal nerve fiber layer (RNFL) thicknesses and optic disc measurements. Associations between post-DST cortisol and ocular measurements were evaluated as secondary analyses. Associations between ACTH concentrations and ocular parameters were evaluated as exploratory analyses (Table S1).

The study was not preregistered, and the statistical analysis plan, including designation of whole-image DCP vessel density as the primary outcome, was finalized after data extraction and before formal statistical analyses. Outcomes and analyses were organized hierarchically. The primary analysis was the adjusted comparison of whole-image DCP vessel density between the MACS and NFA groups. Secondary analyses comprised the adjusted group comparisons of the secondary outcomes listed above and the models in which ln(post-DST cortisol) was entered as a continuous exposure. Analyses of the exploratory macular parameters and ACTH, tertile analyses, models using untransformed post-DST cortisol, and all sensitivity analyses were exploratory. Secondary and exploratory findings are interpreted as hypothesis-generating.

### 2.6. Statistical Analysis

The distribution of continuous variables was evaluated using the Shapiro–Wilk test. Normally distributed variables are presented as mean ± standard deviation, non-normally distributed variables as median and interquartile range, and categorical variables as number and percentage.

Comparisons between the MACS and NFA groups were performed using the independent-sample *t*-test or Mann–Whitney U test, as appropriate. Categorical variables were compared using the Pearson chi-square test or Fisher’s exact test.

The primary adjusted analysis was performed using multivariable linear regression, with whole-image DCP vessel density as the dependent variable. The model was adjusted for age, sex, body mass index, hypertension, diabetes mellitus, hyperlipidemia, and cardiovascular disease. These covariates were selected on clinical grounds as factors potentially related to retinal vessel density and RNFL thickness; the cardiometabolic comorbidities are also more prevalent in MACS than in NFA [1,2]. Because hypertension, diabetes mellitus, hyperlipidemia, and cardiovascular disease may partly mediate the effects of cortisol excess, models adjusted for age and sex only, and for age, sex, and body mass index, were fitted as sensitivity analyses. Results are presented as regression coefficients (β), 95% confidence intervals (CIs), and *p* values.

Because post-DST cortisol concentrations showed a right-skewed distribution, they were natural-log transformed for continuous analyses. Associations between ln-transformed post-DST cortisol and ocular parameters were evaluated using multivariable linear regression. Post-DST cortisol tertiles were examined as exploratory analyses. Unadjusted ordered differences across tertiles were assessed using the Jonckheere–Terpstra test, and adjusted linear trends were evaluated by entering the tertile as an ordinal variable in the regression models.

Because the study had a single designated primary outcome, no multiplicity adjustment was applied to the primary analysis. For secondary and exploratory analyses, the Benjamini–Hochberg false discovery rate procedure was applied separately within each analysis family: unadjusted between-group comparisons, adjusted between-group models, ln-transformed post-DST cortisol models, ACTH models, and adjusted tertile-trend analyses. Each family comprised 27 ocular outcomes. Although the whole-image DCP comparison was included in the between-group adjustment calculations, inference for the primary comparison was based on its unadjusted *p* value. False-discovery-rate-adjusted *p* values are reported as q values. All tests were two-sided; *p* < 0.05 was considered nominally significant, and q < 0.05 indicated statistical significance after false discovery rate correction.

Missing values were not imputed in the main analyses; each model included the patients with complete data for all variables in that model (complete-case analysis), so the number of patients varied between models. Sensitivity analyses examined alternative covariate sets, both in all available patients and in the complete-case sample of the main models (Table S2), and alternative analysis populations (exclusion of six patients with at least one potentially implausible OCTA or OCT value identified during data-quality review, right eyes only, and women only), together with multiple imputation by chained equations for missing values among variables included in the imputation model in participants with an observed outcome. In the continuous-exposure analyses, missing post-DST cortisol values were also imputed. Two hundred imputations were generated using predictive mean matching, and estimates were pooled using Rubin’s rules (Table S3). Patients without the relevant outcome were excluded before imputation. For superior RNFL thickness, additional analyses included adjustment for optic disc area, robust regression with a Huber M-estimator, exclusion of observations with an absolute studentized residual greater than 3, and Bonferroni correction across the 27 outcomes of each analysis family (Table S4). Because of the retrospective design, no a priori sample-size calculation was performed; all eligible patients evaluated during the study period were included. Statistical analyses were performed using IBM SPSS Statistics version 26.0 (IBM Corp., Armonk, NY, USA). Multiple imputation and robust regression were performed in Python 3.11 using statsmodels 0.15.

## 3. Results

### 3.1. Study Population and Clinical Characteristics

The study included 98 patients with adrenal incidentalomas, comprising 45 patients with nonfunctioning adrenal adenomas (NFA) and 53 patients with mild autonomous cortisol secretion (MACS). OCTA measurements were available for 89 participants. The demographic, cardiometabolic, and endocrine characteristics of the study population are summarized in Table 1. The unadjusted between-group ocular comparisons are presented in Table 2.

Of the nine patients without evaluable OCTA (four NFA and five MACS), OCTA had not been performed or did not meet the image-quality criteria. Structural OCT RNFL measurements were available for 91 patients and RPC measurements for 83–87. Among the 89 patients with evaluable OCTA, body mass index was missing in nine and comorbidity data in three (ten patients with at least one missing covariate), and a numerical post-DST cortisol value was missing in two; the adjusted models shown in Table 3 therefore included 74–81 patients. Patients without evaluable OCTA did not differ from those with evaluable imaging in age, sex, group distribution, post-DST cortisol, body mass index, or comorbidities (all *p* ≥ 0.11), although their adenomas were larger (median, 33.0 vs. 25.0 mm; *p* = 0.048). Patients not included in the complete-case primary model were more often male (4 of 19 vs. 3 of 79; *p* = 0.025) but were otherwise similar (all *p* ≥ 0.07).

*p* values are from independent-sample *t*-tests, Mann–Whitney U tests, chi-square tests or Fisher’s exact tests, as appropriate, and are presented descriptively without multiplicity adjustment. Group assignment followed the final endocrinologist classification; 1 mg DST cortisol is shown descriptively.

Compared with the NFA group, the MACS group had higher frequencies of diabetes mellitus, hyperlipidemia, and cardiovascular disease. Plasma ACTH was lower in patients with MACS than in those with NFA (median, 8.0 [interquartile range, 5.8–14.0] vs. 14.0 [10.0–16.8] pg/mL; *p* = 0.002) and was below 10 pg/mL in 32 of 52 (61.5%) and 6 of 30 (20.0%) patients, respectively (*p* < 0.001). In all 26 patients with MACS in whom a 4 mg dexamethasone suppression test was available, cortisol remained above 1.8 µg/dL (median, 2.4 µg/dL; range, 1.87–5.30 µg/dL).

### 3.2. Primary Outcome and Between-Group Ocular Findings

The primary outcome, whole-image deep capillary plexus vessel density (DCP-VD), was 48.64 ± 5.71% in the NFA group and 47.31 ± 5.77% in the MACS group (*p* = 0.279; Table 2). No superficial capillary plexus measurement differed between the groups in any sector (whole image, *p* = 0.835; parafovea, *p* = 0.853; perifovea, *p* = 0.872; fovea, *p* = 0.624).

**Table 2:** OCTA and OCT parameters: unadjusted between-group comparison.

Parameter	NFA	MACS	*p*	*p* (FDR)
DCP vessel density, whole image, % (primary)	48.64 ± 5.71	47.31 ± 5.77	0.279	-
DCP vessel density, parafovea, %	54.24 ± 4.49	52.26 ± 5.36	0.064	0.435
DCP vessel density, fovea, %	36.54 ± 9.03	34.29 ± 7.55	0.203	0.785
DCP vessel density, perifovea, %	49.56 ± 6.63	48.34 ± 6.28	0.375	0.845
SCP vessel density, whole image, %	50.09 ± 4.13	49.91 ± 3.88	0.835	0.979
SCP vessel density, parafovea, %	52.10 (47.60–57.00)	52.85 (51.15–54.70)	0.853	0.979
SCP vessel density, fovea, %	18.20 (13.20–21.60)	18.30 (12.95–22.73)	0.624	0.979
SCP vessel density, perifovea, %	50.79 ± 4.23	50.65 ± 3.93	0.872	0.979
SCP/DCP vessel density ratio	1.004 (0.971–1.096)	1.053 (0.961–1.175)	0.513	0.979
FAZ area, mm^2^	0.298 ± 0.111	0.300 ± 0.109	0.913	0.979
FAZ perimeter, mm	2.209 (1.893–2.352)	2.133 (1.921–2.456)	0.867	0.979
FAZ acircularity index	1.090 (1.073–1.108)	1.095 (1.082–1.129)	0.065	0.435
FAZ circularity index	0.842 (0.814–0.868)	0.834 (0.785–0.855)	0.065	0.435
FD vessel density, %	55.01 (50.50–58.26)	53.83 (48.88–56.40)	0.198	0.785
Outer retinal flow area, mm^2^	0.807 (0.502–1.454)	0.688 (0.449–1.228)	0.639	0.979
RPC vessel density, whole image, %	50.36 ± 2.59	50.37 ± 2.26	0.979	0.979
RPC vessel density, inside disc, %	49.45 (45.80–53.88)	48.50 (45.30–50.90)	0.152	0.785
RPC vessel density, peripapillary, %	54.40 (50.45–55.55)	52.80 (51.45–54.78)	0.593	0.979
RNFL thickness, average, µm	106.7 ± 12.5	109.5 ± 11.9	0.260	0.836
RNFL thickness, superior, µm	120.3 ± 22.3	130.7 ± 16.0	0.012	0.316
RNFL thickness, inferior, µm	132.7 ± 21.5	136.5 ± 18.7	0.358	0.845
RNFL thickness, nasal, µm	102.7 ± 14.0	102.6 ± 16.4	0.968	0.979
RNFL thickness, temporal, µm	71.6 ± 13.0	70.5 ± 11.1	0.673	0.979
Disc area, mm^2^	1.915 (1.650–2.155)	1.890 (1.660–2.205)	0.951	0.979
Rim area, mm^2^	1.765 (1.512–1.982)	1.810 (1.590–1.990)	0.879	0.979
Cup/disc area ratio	0.000 (0.000–0.107)	0.030 (0.000–0.130)	0.320	0.845
Cup volume, mm^3^	0.000 (0.000–0.021)	0.004 (0.000–0.030)	0.433	0.900

OCTA-evaluable subset: NFA *n* = 39–43 and MACS *n* = 44–48, depending on the outcome. The primary outcome is listed first; remaining outcomes are secondary.

*p* (FDR) = Benjamini–Hochberg adjusted *p* value across the 27 comparisons. SCP = superficial capillary plexus; DCP = deep capillary plexus; RPC = radial peripapillary capillary; FAZ = foveal avascular zone; RNFL = retinal nerve fiber layer; FD = vessel density in the 300 µm ring around the FAZ.

After adjustment for age, sex, body mass index, hypertension, diabetes mellitus, hyperlipidemia, and cardiovascular disease, the between-group difference remained nonsignificant (β = −1.29 percentage points; 95% confidence interval [CI], −3.97 to 1.38; *p* = 0.339; Table 3).

**Table 3:** Primary and selected secondary multivariable regression analyses.

Outcome	Exposure	β	95% CI	*p*	q	*n*
DCP vessel density, whole image, % (primary outcome)	MACS vs. NFA	−1.29	−3.97 to 1.38	0.339	-	79
DCP vessel density, parafovea, %	MACS vs. NFA	−2.256	−4.662 to 0.150	0.066	0.307	79
RPC vessel density, inside disc, %	MACS vs. NFA	−1.162	−4.463 to 2.139	0.485	0.935	75
RNFL thickness, average, µm	MACS vs. NFA	6.309	0.665 to 11.953	0.029	0.307	81
RNFL thickness, superior, µm	MACS vs. NFA	17.379	9.536 to 25.222	<0.001	<0.001	81
FAZ acircularity index	MACS vs. NFA	0.032	−0.002 to 0.067	0.068	0.307	78
DCP vessel density, whole image, %	ln(1 mg DST cortisol), per unit	−2.242	−4.649 to 0.164	0.067	0.303	77
DCP vessel density, parafovea, %	ln(1 mg DST cortisol), per unit	−2.716	−4.845 to −0.587	0.013	0.178	77
RPC vessel density, inside disc, %	ln(1 mg DST cortisol), per unit	−3.129	−5.875 to −0.383	0.026	0.236	74
RNFL thickness, average, µm	ln(1 mg DST cortisol), per unit	4.150	−1.042 to 9.342	0.115	0.346	80
RNFL thickness, superior, µm	ln(1 mg DST cortisol), per unit	13.738	6.442 to 21.035	<0.001	0.010	80
FAZ acircularity index	ln(1 mg DST cortisol), per unit	0.030	0.0003 to 0.0603	0.048	0.259	76

The primary outcome is shown irrespective of statistical significance. Six parameters are shown for both exposures, irrespective of statistical significance. Post-dexamethasone cortisol is modeled on the natural-log scale. The maximum variance inflation factor across all models was 1.75. Each row represents a separate multivariable linear regression model adjusted for age, sex, body mass index, hypertension, diabetes mellitus, hyperlipidemia, and cardiovascular disease. β represents the adjusted MACS–NFA mean difference or the adjusted change in the outcome per one-unit increase in ln(post-DST cortisol). q values were calculated using the Benjamini–Hochberg procedure separately across all 27 between-group models and all 27 ln(post-DST cortisol) models, including outcomes not displayed in this table. The primary comparison was interpreted using its *p* value without multiplicity adjustment; its q value is therefore not displayed. CI, confidence interval; DCP, deep capillary plexus; RPC, radial peripapillary capillary; RNFL, retinal nerve fiber layer; FAZ, foveal avascular zone; DST, dexamethasone suppression test.

**Figure 1:**
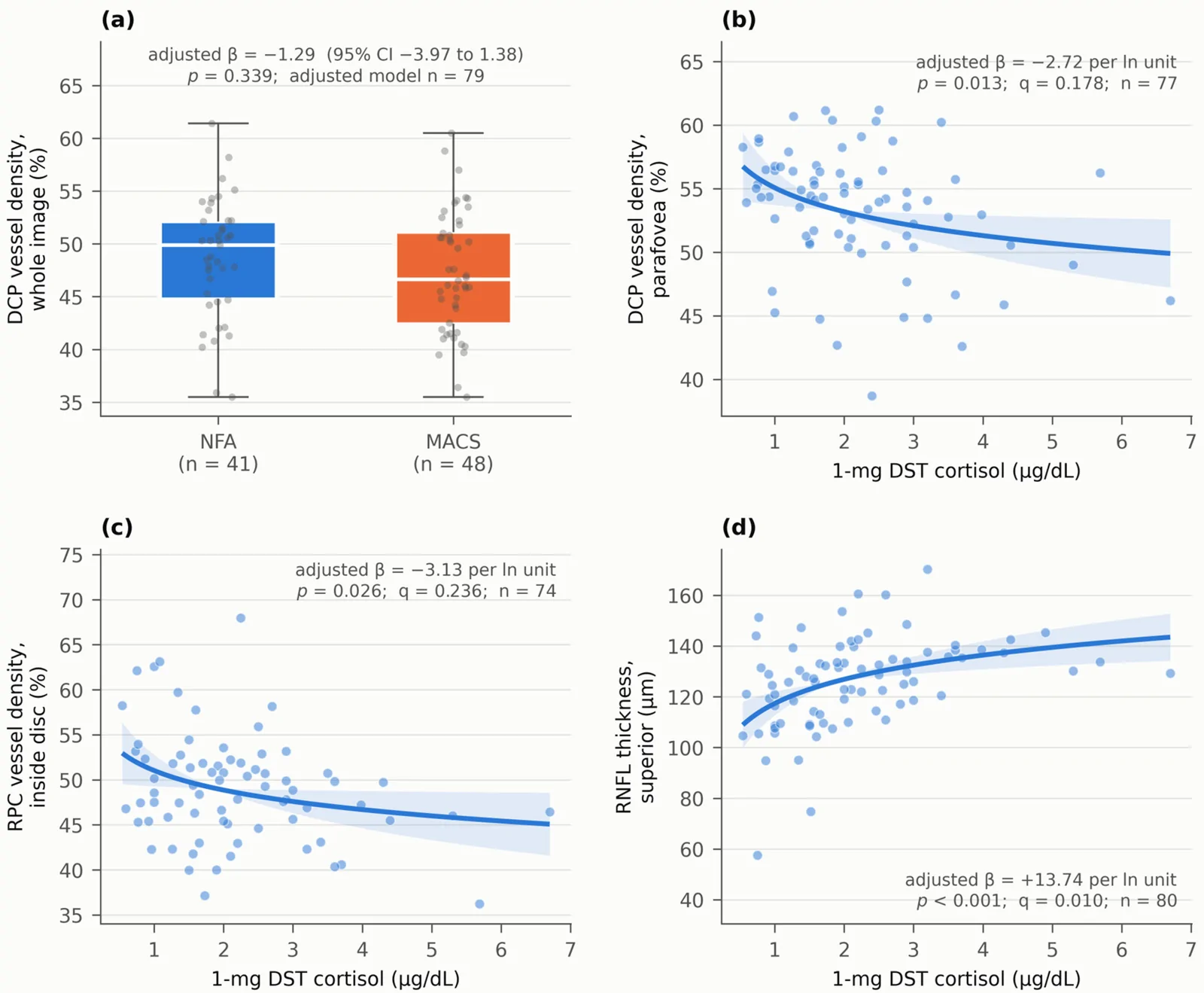
Autonomous cortisol secretion and retinal microvascular parameters. (**a**) Deep capillary plexus (DCP) vessel density, whole image—the primary outcome—in patients with a non-functioning adrenal adenoma (NFA) and with mild autonomous cortisol secretion (MACS). Blue and orange boxes represent the NFA and MACS groups, respectively. Boxes show the median and interquartile range, whiskers extend to 1.5 × IQR and grey points are individual patients; all available primary measurements are displayed (NFA *n* = 41, MACS *n* = 48). The annotated estimate is the between-group difference adjusted for age, sex, body mass index, hypertension, diabetes mellitus, hyperlipidemia and cardiovascular disease, obtained from the complete-case multivariable model (*n* = 79). (**b**) DCP vessel density, parafovea; (**c**) radial peripapillary capillary (RPC) vessel density inside the disc; and (**d**) superior peripapillary retinal nerve fiber layer (RNFL) thickness, each plotted against 1 mg dexamethasone-suppressed serum cortisol. In panels (**b**–**d**), points represent partial residuals from the multivariable models, recentered on the observed outcome mean. The fitted relationship is linear in ln(post-DST cortisol) and is displayed on the original cortisol scale. β represents the adjusted change in the outcome per one-unit increase in ln(post-DST cortisol). The exposure was modelled on the natural-logarithmic scale and is displayed on the untransformed axis; the line is the adjusted model prediction, and the shaded band its 95% confidence interval. β, adjusted regression coefficient (MACS versus NFA in panel (**a**); per one unit of ln-transformed post-dexamethasone cortisol in panels (**b**–**d**)); q, Benjamini–Hochberg false discovery rate adjusted *p*-value across the 27 ocular outcomes tested for the same exposure. No multiplicity adjustment was applied to the primary outcome in panel (**a**).

In the multivariable analysis, superior RNFL thickness was greater in patients with MACS than in those with NFA (β = 17.38 µm; 95% CI, 9.54–25.22; *p* < 0.001). This was the only adjusted between-group ocular finding that remained significant after false discovery rate correction (q < 0.001). The distribution of whole-image DCP-VD in the NFA and MACS groups is shown in Figure 1a.

### 3.3. Associations with Cortisol Nonsuppression

When post-DST cortisol was analyzed as a continuous log-transformed variable, increasing ln(DST) was associated with lower parafoveal DCP-VD (β = −2.72 percentage points; 95% CI, −4.85 to −0.59; *p* = 0.013), lower inside-disc radial peripapillary capillary vessel density (β = −3.13 percentage points; 95% CI, −5.88 to −0.38; *p* = 0.026), and greater superior RNFL thickness (β = 13.74 µm; 95% CI, 6.44–21.04; *p* < 0.001). A nominal association was also observed with the foveal avascular zone acircularity index (*p* = 0.048).

After false discovery rate correction, only the association between ln(DST) and superior RNFL thickness remained statistically significant (q = 0.010). The associations with parafoveal DCP-VD, inside-disc radial peripapillary capillary vessel density, and foveal avascular zone acircularity did not retain statistical significance.

The direction of the associations was similar in analyses using untransformed post-DST cortisol, although nominal statistical significance varied between models (Table 4). The relationships of post-DST cortisol with parafoveal DCP-VD, inside-disc RPC-VD, and superior RNFL thickness are illustrated in Figure 1b–d.

The same six ocular outcomes presented in Table 3 are shown irrespective of statistical significance. *p* values are nominal, and these sensitivity analyses are interpreted as supportive rather than confirmatory.

### 3.4. Tertile and Sensitivity Analyses

Superior RNFL thickness increased across increasing DST tertiles (122.19 ± 23.17, 122.57 ± 18.93, and 133.14 ± 15.40 µm; adjusted *p* for trend = 0.001; Table 5), and this trend remained significant after false discovery rate correction (q = 0.038). Parafoveal DCP-VD and inside-disc radial peripapillary capillary vessel density decreased numerically across increasing DST tertiles; however, these trends did not remain statistically significant after correction for multiple comparisons.

Overall, categorical MACS status was not associated with whole-image DCP-VD, whereas increasing cortisol nonsuppression revealed exploratory regional microvascular signals. Superior RNFL thickness was the only ocular finding that remained consistently significant after correction for multiple comparisons.

Exploratory associations between ACTH concentrations and ocular parameters are presented in Supplementary Table S1. None remained statistically significant after false discovery rate correction.

In additional sensitivity analyses, the primary outcome was not associated with MACS status in any covariate specification or analysis population, or after multiple imputation of missing covariates (*p* = 0.107–0.447; Tables S2 and S3). When fitted in the same patients as the main models, estimates adjusted for age and sex only, or for age, sex, and body mass index, were close to the fully adjusted estimates; in all available patients, the age- and sex-adjusted estimates were directionally unchanged, with a smaller but still significant association for superior RNFL thickness (Table S2). The continuous associations with parafoveal DCP-VD and superior RNFL thickness remained nominally significant in every analysis population and after multiple imputation, whereas the inside-disc RPC association remained nominally significant in women and after multiple imputation but not after exclusion of implausible values or restriction to right eyes (both *p* = 0.08; Table S3). Results restricted to women were virtually identical to those of the whole cohort. The superior RNFL association was robust to adjustment for optic disc area, robust regression, and exclusion of outlying values. In the main models, it remained significant after Bonferroni correction across the 27 outcomes of each analysis family (adjusted *p* = 0.001 for the group comparison and *p* = 0.010 for ln[post-DST cortisol]). The inferior quadrant showed a directionally similar but nonsignificant association, whereas estimates for the nasal and temporal quadrants were close to zero and nonsignificant (all *p* ≥ 0.62; Table S4).

## 4. Discussion

In this cross-sectional study, the primary outcome, whole-image DCP-VD, did not differ significantly between patients with MACS and those with NFA, and this null result was consistent across all sensitivity analyses; it is the principal finding of the study. In secondary analyses, higher post-DST cortisol was associated with lower parafoveal DCP and inside-disc RPC vessel density and with greater superior RNFL thickness, but not with any superficial capillary plexus measurement, and only the RNFL associations survived correction for multiple testing. Because exposure and outcomes were measured during a single diagnostic work-up, these associations describe co-variation; they cannot show whether cortisol excess precedes the retinal findings, whether the relationship runs in the opposite direction, or whether both reflect a shared underlying factor. The secondary findings are therefore hypothesis-generating.

MACS is increasingly viewed as a continuum rather than a strictly binary state, and the current European guideline recommends interpreting post-DST cortisol within its clinical context [1]. Compared with NFA, MACS is associated with more hypertension, diabetes, dyslipidemia, and cardiovascular disease [1,2], higher post-DST cortisol has been associated with increased mortality [3], and adrenalectomy may improve cardiometabolic outcomes in selected patients [14]. Ocular evidence comes mainly from overt Cushing syndrome, in which choroidal thickening, pachychoroid features, and central serous chorioretinopathy have been described [7,8,15] in the setting of more pronounced hypercortisolism than is typically seen in MACS.

The available ocular studies of mild cortisol excess, including ours, do not show a consistent pattern. One study reported greater subfoveal choroidal thickness and more frequent pachychoroid features in MACS than in NFA [9]; a pilot study found no choroidal or choriocapillaris differences between subclinical Cushing syndrome and healthy controls and no correlation with any hormonal parameter [10]; and a conference abstract described higher superficial and deep plexus vessel density, higher choriocapillaris perfusion, and a smaller FAZ in 51 patients analyzed as 102 eyes [11]. These studies differ in comparator, anatomical target, handling of fellow eyes, and covariate adjustment, and the two studies that assessed the choriocapillaris reported different findings [10,11], one of which is available only as a conference abstract [11]. This discordance may partly reflect differences in study design, populations, and imaging methods.

The regional DCP and RPC associations are biologically plausible but exploratory. In animal models, glucocorticoid excess caused endothelial cell death and capillary rarefaction in nonocular vascular beds [4,5], and cortisol may also act indirectly through hypertension, dysglycemia, and dyslipidemia. The estimates were similar when the models were adjusted for age and sex only, but residual confounding cannot be excluded. OCTA vessel density is a flow-dependent imaging metric rather than a histological capillary count [6]; lower values may reflect reduced perfusion, flow below the detection threshold, anatomical variation, or technical factors and should not be interpreted as capillary loss. Neither regional association survived multiplicity correction.

Superior RNFL thickness was the most robust secondary finding: it was consistent across categorical, continuous, and tertile analyses and across all sensitivity analyses (Table S4), and in the main models it remained significant after false discovery rate and Bonferroni correction. This argues against multiplicity alone as an explanation, although consistency within a single dataset is not replication. The direction of this association was not anticipated, and its biological meaning is uncertain; greater RNFL thickness should not be interpreted as a favorable structural change. Three explanations merit consideration. First, measurement factors: signal strength influences quantitative OCTA measurements [16], and axial length affects OCTA magnification [17] and measured RNFL thickness [18], so systematic between-group differences in either could produce artifactual differences. Neither was available. Optic disc area, which is also magnification-dependent, was unrelated to cortisol exposure, and adjustment for it did not alter the estimate; however, this does not fully account for axial-length-related effects. Second, peripapillary RNFL values include the major retinal vessels, whose contribution is greatest in the superior and inferior sectors [19]; the association was significant only in the superior quadrant, directionally similar but nonsignificant in the inferior quadrant, and close to zero nasally and temporally, so differences in vessel caliber or position may contribute. Third, in an experimental model, mineralocorticoid receptor activation upregulated ion and water channels in retinal Müller glia [20]; if a similar mechanism operated in humans, subtle changes in neuroretinal fluid handling could increase measured thickness. None of these possibilities can be tested with the present data, and the finding requires confirmation with axial length correction and image-quality metrics.

The strengths of this study include guideline-based endocrine classification supported by ACTH measurements and repeat dexamethasone testing when indicated; ocular imaging performed during the initial diagnostic work-up, before dexamethasone administration and before any adrenal-directed or cortisol-lowering treatment, and therefore before the DST-based classification was available; an NFA comparator that shares the adrenal background and helps distinguish the effects of cortisol autonomy from those of an adrenal mass; the analysis of one eye per patient; a single primary outcome reported first irrespective of its result; complementary categorical and continuous modeling of cortisol exposure; and extensive sensitivity analyses.

This study has limitations. The retrospective, single-center, cross-sectional design precludes causal or temporal inference, and apart from the primary comparison, the analyses should be regarded as exploratory. Healthy controls were not included; therefore, we cannot determine whether patients with NFA themselves differ from the general population. The cohort was 93% female; results restricted to women were virtually identical, but the findings may not apply to men. OCTA was unavailable in nine patients, and covariate data were incomplete in ten others. Patients without evaluable OCTA had larger adenomas, and those not included in the complete-case primary model were more often male; after multiple imputation, the primary outcome remained nonsignificant and the continuous associations with parafoveal DCP-VD, inside-disc RPC vessel density, and superior RNFL thickness remained nominally significant (Table S3). Nevertheless, selection bias cannot be excluded. Serum dexamethasone concentrations were not measured, so some misclassification of cortisol status is possible. Axial length and individual signal-strength indices were unavailable [16,17]; refractive error was restricted to ±3.00 D, but magnification and image-quality effects cannot be excluded. Adjustment for hypertension, diabetes, hyperlipidemia, and cardiovascular disease could remove part of a genuine association if these conditions mediate the effects of cortisol [21], although parsimonious models gave similar estimates. Finally, repeat OCTA acquisitions were not available, so measurement repeatability could not be assessed.

At present, whole-image DCP-VD cannot be recommended as a marker for distinguishing MACS from NFA, and OCTA should be regarded as a research tool in this setting. Prospective multicenter studies that include healthy controls, NFA, and functioning adenomas, use standardized acquisition with image-quality indices and axial length correction, and ideally examine patients before and after adrenalectomy are needed to establish whether these associations are reproducible and whether they change as cortisol exposure decreases.

## 5. Conclusions

In this cross-sectional study, MACS was not significantly associated with whole-image DCP vessel density, the primary outcome. In secondary analyses, higher post-DST cortisol was associated with lower regional DCP and RPC vessel density and greater superior RNFL thickness. Only the RNFL associations survived multiplicity correction, and even these should be interpreted cautiously in the absence of axial length and individual signal-strength data. All secondary associations are hypothesis-generating and do not establish ocular involvement in MACS. Prospective studies with standardized imaging, axial length correction, and assessment before and after treatment are needed to determine whether they are reproducible and clinically meaningful.

## Figures and Tables

**Table 1:** Demographic, clinical, and endocrine characteristics of the study population.

Variable	NFA	MACS	*p*
Female sex, *n* (%)	43 (95.6)	48 (90.6)	0.447
Bilateral adenoma, *n* (%)	9 (20.9)	15 (28.8)	0.518
Diabetes mellitus, *n* (%)	5 (11.4)	16 (31.4)	0.036
Hypertension, *n* (%)	17 (38.6)	22 (43.1)	0.814
Hyperlipidaemia, *n* (%)	13 (29.5)	29 (56.9)	0.014
Cardiovascular disease, *n* (%)	1 (2.3)	8 (15.7)	0.035
Age, years	54.0 (46.0–59.0)	56.0 (50.0–60.0)	0.128
Body mass index, kg/m^2^	28.7 (25.5–31.6)	30.5 (27.5–32.9)	0.135
1 mg DST cortisol, µg/dL	1.14 (0.85–1.53)	2.65 (2.19–3.20)	<0.001

Data are presented as *n* (%), mean ± SD or median (IQR), as appropriate. The study included 45 patients with NFA and 53 with MACS; denominators vary because of missing data.

**Table 4:** Sensitivity analysis using untransformed 1 mg DST cortisol.

Outcome	β per µg/dL	95% CI	*p*	*n*
RNFL thickness, superior, µm	5.492	2.169 to 8.815	0.002	80
DCP vessel density, parafovea, %	−1.254	−2.203 to −0.304	0.010	77
RPC vessel density, inside disc, %	−1.446	−2.675 to −0.217	0.022	74
DCP vessel density, whole image, %	−1.094	−2.166 to −0.023	0.045	77
RNFL thickness, average, µm	1.468	−0.866 to 3.802	0.214	80
FAZ acircularity index	0.012	−0.001 to 0.026	0.080	76

The same adjusted models were fitted with 1 mg DST cortisol entered without log transformation. These are supporting analyses and do not replace the group-based primary analysis.

**Table 5:** OCTA and OCT parameters across tertiles of 1 mg DST cortisol.

Parameter	T1	T2	T3	*p* (J–T)	*p* for Trend *
RNFL thickness, superior, µm	122.19 ± 23.17	122.57 ± 18.93	133.14 ± 15.40	0.024	0.001
FAZ circularity index	0.83 ± 0.05	0.83 ± 0.07	0.79 ± 0.10	0.078	0.053
FAZ acircularity index	1.10 ± 0.04	1.10 ± 0.06	1.13 ± 0.09	0.078	0.053
DCP vessel density, parafovea, %	54.25 ± 4.60	53.65 ± 4.98	51.70 ± 5.21	0.031	0.068
RPC vessel density, inside disc, %	51.40 ± 6.43	49.06 ± 7.78	46.83 ± 4.72	0.011	0.081
RNFL thickness, average, µm	107.32 ± 14.19	106.13 ± 11.07	111.14 ± 10.92	0.330	0.087
RNFL thickness, inferior, µm	134.06 ± 22.73	129.87 ± 19.10	140.07 ± 17.36	0.314	0.138
FAZ area, mm^2^	0.30 ± 0.11	0.31 ± 0.10	0.29 ± 0.12	0.654	0.236
Outer retinal flow area, mm^2^	1.01 ± 0.60	0.86 ± 0.56	0.86 ± 0.53	0.459	0.236
SCP/DCP vessel density ratio	1.04 ± 0.12	1.04 ± 0.13	1.09 ± 0.17	0.520	0.357
FAZ perimeter, mm	2.08 ± 0.40	2.14 ± 0.46	2.09 ± 0.47	0.868	0.462
DCP vessel density, whole image, %	48.55 ± 5.79	48.43 ± 5.68	46.83 ± 5.86	0.260	0.481

T1 < 1.51 µg/dL; T2 1.51 to <2.47 µg/dL; T3 ≥ 2.47 µg/dL. The 96 participants with an available numeric DST value were divided into three groups of 32; outcome-specific *n* varied because of missing imaging data. Values are mean ± SD. *p* (J–T) = Jonckheere–Terpstra test for ordered alternatives (unadjusted). * Adjusted *p* for trend obtained by entering tertile rank as a continuous term in the multivariable model. Tertile analyses are exploratory.

## Data Availability

The data supporting the findings of this study are not publicly available because of patient privacy and ethical restrictions. De-identified data may be made available by the corresponding author upon reasonable request and subject to institutional and Ethics Committee approval.

## Supplementary Materials

The following supporting information can be downloaded at: https://www.mdpi.com/article/10.3390/jcm15207850/s1, Table S1: Exploratory associations between ACTH concentrations and ocular parameters; Table S2: Sensitivity of the primary outcome and principal secondary associations to covariate specification; Table S3: Sensitivity of the primary outcome and principal secondary associations to the analysis population and to missing data; Table S4: Robustness of the associations of MACS status and post-DST cortisol with superior RNFL thickness.

## Abbreviations

The following abbreviations are used in this manuscript:

ACTH	Adrenocorticotropic hormone
BMI	Body mass index
CI	Confidence interval
DCP	Deep capillary plexus
DST	Dexamethasone suppression test
FAZ	Foveal avascular zone
FD	Vessel density in the 300 µm ring surrounding the foveal avascular zone
FDR	False discovery rate
MACS	Mild autonomous cortisol secretion
NFA	Nonfunctioning adrenal adenoma
OCT	Optical coherence tomography
OCTA	Optical coherence tomography angiography
RNFL	Retinal nerve fiber layer
RPC	Radial peripapillary capillary
SCP	Superficial capillary plexus
VD	Vessel density
