# Supplementary material for: Retinal Microvascular and Peripapillary Profiles in Mild Autonomous Cortisol Secretion Versus Nonfunctioning Adrenal Adenomas
Source: J Clin Med. 2026 Oct 12;15(20):7850. doi: 10.3390/jcm15207850 (PMC13705467; doi:10.3390/jcm15207850)
Supplement: Supplementary file 1 [file jcm-15-07850-s001.zip › jcm-4580446-supplementary.pdf]

Supplementary Materials

Table S1. Exploratory associations between ACTH concentrations and ocular parameters.

Outcome	β per pg/mL	95% CI	p	q	n
DCP vessel density, whole image, %	−0.0882	−0.2320 to 0.0556	0.225	0.414	68
DCP vessel density, parafovea, %	−0.1125	−0.2411 to 0.0161	0.085	0.329	68
DCP vessel density, fovea, %	0.1392	−0.0703 to 0.3486	0.189	0.414	68
DCP vessel density, perifovea, %	−0.0989	−0.2611 to 0.0632	0.227	0.414	68
SCP vessel density, whole image, %	0.0613	−0.0444 to 0.1670	0.251	0.423	68
SCP vessel density, parafovea, %	0.0500	−0.1032 to 0.2032	0.516	0.732	68
SCP vessel density, fovea, %	0.1554	−0.0659 to 0.3767	0.165	0.414	68
SCP vessel density, perifovea, %	0.0640	−0.0400 to 0.1681	0.223	0.414	68
SCP/DCP vessel density ratio	0.0036	0.0002 to 0.0070	0.038	0.209	68
FAZ area, mm²	−0.0043	−0.0070 to −0.0015	0.003	0.074	67
FAZ perimeter, mm	−0.0148	−0.0265 to −0.0030	0.014	0.130	67
FAZ acircularity index	0.0012	−0.0007 to 0.0031	0.222	0.414	67
FAZ circularity index	−0.0014	−0.0037 to 0.0009	0.230	0.414	67
FD, %	−0.1495	−0.2910 to −0.0079	0.039	0.209	67
Outer retinal flow area, mm²	0.0122	−0.0029 to 0.0272	0.112	0.378	68
RPC vessel density, whole image, %	−0.0764	−0.1349 to −0.0179	0.011	0.130	66
RPC vessel density, inside disc, %	−0.0073	−0.1729 to 0.1583	0.930	0.930	65
RPC vessel density, peripapillary, %	−0.0782	−0.1552 to −0.0013	0.047	0.209	63
RNFL thickness, average, μm	−0.0266	−0.3281 to 0.2748	0.860	0.893	69
RNFL thickness, superior, μm	−0.2584	−0.7187 to 0.2020	0.266	0.423	69
RNFL thickness, inferior, μm	−0.1263	−0.5923 to 0.3396	0.590	0.732	69
RNFL thickness, nasal, μm	0.1262	−0.2781 to 0.5305	0.535	0.732	69
RNFL thickness, temporal, μm	0.0833	−0.2288 to 0.3955	0.595	0.732	68
Disc area, mm²	0.0017	−0.0093 to 0.0127	0.757	0.817	67
Rim area, mm²	0.0025	−0.0068 to 0.0117	0.596	0.732	67
Cup/disc area ratio	−0.0005	−0.0031 to 0.0021	0.693	0.808	67
Cup volume, mm³	−0.0002	−0.0013 to 0.0009	0.719	0.808	67

Each row represents a separate multivariable linear regression model adjusted for age, sex, body mass index, hypertension, diabetes mellitus, hyperlipidemia, and cardiovascular disease. β represents the adjusted change in the outcome per 1 pg/mL increase in ACTH. q values were calculated using the Benjamini–Hochberg procedure across all 27 ACTH models. Sample sizes varied because of missing data. None of the associations remained statistically significant after false discovery rate correction. ACTH, adrenocorticotropic hormone; CI, confidence interval; DCP, deep capillary plexus; SCP, superficial capillary plexus; FAZ, foveal avascular zone; FD, vessel density in the 300-μm ring surrounding the FAZ; RPC, radial peripapillary capillary; RNFL, retinal nerve fiber layer.

Table S2. Sensitivity of the primary outcome and principal secondary associations to covariate specification.

Outcome	Age + sex, all available	Age + sex, CC	Age + sex + BMI, CC	Main model without HL and CVD	Main model	Main model + adenoma size	Main model + fasting glucose
MACS vs. NFA							
Whole-image DCP VD, % (primary outcome)	−1.16 (0.356)	−1.18 (0.364)	−1.16 (0.381)	−1.05 (0.431)	−1.29 (0.339)	−1.61 (0.257)	−1.29 (0.349)
Parafoveal DCP VD, %	−1.80 (0.102)	−2.11 (0.063)	−2.15 (0.063)	−2.17 (0.066)	−2.26 (0.066)	−2.74 (0.033)	−2.22 (0.076)
Inside-disc RPC VD, %	−1.57 (0.315)	−0.79 (0.644)	−1.41 (0.388)	−1.60 (0.335)	−1.16 (0.485)	−1.60 (0.351)	−1.39 (0.409)
Superior RNFL thickness, μm	11.75 (0.005)	14.26 (<0.001)	15.28 (<0.001)	15.64 (<0.001)	17.38 (<0.001)	15.58 (<0.001)	17.97 (<0.001)
Whole-image SCP VD, %	−0.09 (0.920)	−0.14 (0.886)	0.03 (0.976)	0.13 (0.895)	0.36 (0.713)	0.56 (0.590)	0.17 (0.859)
ln(post-DST cortisol), per unit							
Whole-image DCP VD, % (primary outcome)	−2.03 (0.067)	−2.23 (0.063)	−2.26 (0.061)	−2.13 (0.080)	−2.24 (0.067)	−2.75 (0.038)	−2.23 (0.073)
Parafoveal DCP VD, %	−2.17 (0.024)	−2.64 (0.010)	−2.66 (0.011)	−2.64 (0.013)	−2.72 (0.013)	−3.35 (0.004)	−2.70 (0.015)
Inside-disc RPC VD, %	−3.62 (0.005)	−3.99 (0.005)	−3.65 (0.009)	−3.62 (0.010)	−3.13 (0.026)	−4.27 (0.003)	−3.27 (0.021)
Superior RNFL thickness, μm	9.03 (0.014)	12.82 (<0.001)	12.17 (0.001)	12.41 (0.001)	13.74 (<0.001)	11.47 (0.005)	13.87 (<0.001)
Whole-image SCP VD, %	−0.52 (0.502)	−0.49 (0.588)	−0.60 (0.506)	−0.56 (0.538)	−0.23 (0.793)	−0.21 (0.831)	−0.32 (0.714)

Values are regression coefficients (p). Main model: age, sex, body mass index (BMI), hypertension, diabetes mellitus, hyperlipidemia (HL), and cardiovascular disease (CVD) (n = 74–81). All available: all patients with the outcome and the variables in that model (n = 84–91). CC, complete-case sample: the patients included in the main model for the same outcome and exposure. Main model + fasting glucose: n = 73–80. VD, vessel density; DCP, deep capillary plexus; RPC, radial peripapillary capillary; RNFL, retinal nerve fiber layer; SCP, superficial capillary plexus; DST, dexamethasone suppression test.

Table S3. Sensitivity of the primary outcome and principal secondary associations to the analysis population and to missing data.

Outcome	Main analysis	Excluding implausible values	Right eyes only	Women only	Multiple imputation
MACS vs. NFA					
Whole-image DCP VD, % (primary outcome)	−1.29 (0.339; 79)	−2.35 (0.107; 73)	−2.31 (0.125; 66)	−1.30 (0.343; 76)	−0.99 (0.447; 89)
Parafoveal DCP VD, %	−2.26 (0.066; 79)	−2.95 (0.029; 73)	−2.96 (0.029; 66)	−2.31 (0.062; 76)	−1.86 (0.113; 89)
Inside-disc RPC VD, %	−1.16 (0.485; 75)	−0.87 (0.615; 70)	−0.85 (0.653; 63)	−1.24 (0.459; 73)	−1.76 (0.251; 85)
Superior RNFL thickness, µm	17.38 (<0.001; 81)	13.70 (<0.001; 76)	15.92 (<0.001; 68)	17.29 (<0.001; 79)	14.26 (<0.001; 91)
Whole-image SCP VD, %	0.36 (0.713; 79)	0.38 (0.718; 73)	0.68 (0.538; 66)	0.35 (0.727; 76)	0.39 (0.665; 89)
ln(post-DST cortisol), per unit					
Whole-image DCP VD, % (primary outcome)	−2.24 (0.067; 77)	−2.83 (0.028; 71)	−3.18 (0.018; 64)	−2.24 (0.069; 75)	−1.91 (0.087; 89)
Parafoveal DCP VD, %	−2.72 (0.013; 77)	−3.24 (0.006; 71)	−3.17 (0.008; 64)	−2.72 (0.014; 75)	−2.26 (0.025; 89)
Inside-disc RPC VD, %	−3.13 (0.026; 74)	−2.49 (0.083; 69)	−2.72 (0.080; 62)	−3.13 (0.027; 72)	−2.72 (0.042; 85)
Superior RNFL thickness, µm	13.74 (<0.001; 80)	10.08 (0.004; 75)	10.56 (0.005; 67)	13.72 (<0.001; 78)	9.71 (0.008; 91)
Whole-image SCP VD, %	−0.23 (0.793; 77)	−0.13 (0.890; 71)	−0.33 (0.740; 64)	−0.23 (0.794; 75)	−0.20 (0.802; 89)

Values are regression coefficient (p; n) from the main model (age, sex, body mass index, hypertension, diabetes mellitus, hyperlipidemia, and cardiovascular disease). Excluding implausible values: six patients with at least one potentially implausible OCTA or OCT value identified during data-quality review were excluded. Women only: sex term omitted. Multiple imputation: chained equations with predictive mean matching (200 imputations), pooled with Rubin's rules; the imputation model included the outcome, the exposure, all covariates, and the other exposure; n is the number of patients with the outcome. In the continuous-exposure analyses, missing post-DST cortisol values were also imputed. Patients without the relevant outcome were excluded before imputation. Abbreviations as in Table S2.

Table S4. Robustness of the associations of MACS status and post-DST cortisol with superior RNFL thickness.

**Analysis**	**MACS vs. NFA: β (95% CI); p; n**	**ln(post-DST cortisol): β (95% CI); p; n**
Main analysis	17.38 (9.54 to 25.22); <0.001; 81	13.74 (6.44 to 21.04); <0.001; 80
Main model + optic disc area	17.10 (8.95 to 25.24); <0.001; 78	13.79 (6.33 to 21.26); <0.001; 77
Robust regression (Huber M-estimator)	15.52 (9.02 to 22.01); <0.001; 81	12.00 (5.59 to 18.41); <0.001; 80
Excluding observations with absolute studentized residual > 3	15.67 (8.48 to 22.86); <0.001; 80	10.95 (4.60 to 17.30); <0.001; 78
Excluding potentially implausible values	13.70 (6.32 to 21.07); <0.001; 76	10.08 (3.31 to 16.85); 0.004; 75
Right eyes only	15.92 (8.02 to 23.82); <0.001; 68	10.56 (3.23 to 17.90); 0.005; 67
Women only	17.29 (9.37 to 25.21); <0.001; 79	13.72 (6.39 to 21.06); <0.001; 78
Age and sex only, all available patients	11.75 (3.72 to 19.78); 0.005; 91	9.03 (1.87 to 16.20); 0.014; 90
Multiple imputation (200 imputations)	14.26 (6.12 to 22.41); <0.001; 91	9.71 (2.49 to 16.92); 0.008; 91
Bonferroni-adjusted p, main analysis	0.001	0.010

β, adjusted difference (MACS vs. NFA) or change per one-unit increase in ln(post-DST cortisol), in μm. Unless stated otherwise, models were adjusted as in the main analysis. Bonferroni correction was applied across the 27 outcomes of the respective analysis family. Maximum Cook's distance in the main models: 0.067 (MACS vs. NFA) and 0.086 (ln cortisol). Other RNFL quadrants in the main model (MACS vs. NFA / ln cortisol): average 6.31 (p = 0.029) / 4.15 (p = 0.115); inferior 9.12 (p = 0.053) / 5.50 (p = 0.202); nasal 0.42 (p = 0.911) / −1.69 (p = 0.624); temporal 0.47 (p = 0.870) / −0.74 (p = 0.773). Optic disc area was not associated with MACS status (β = −0.01 mm²; p = 0.935) or with ln cortisol (β = −0.07 mm²; p = 0.519).
